# Unplanned readmissions in younger and older adult patients: the role of healthcare-related adverse events

**DOI:** 10.1186/s40001-016-0230-0

**Published:** 2016-09-15

**Authors:** Fabienne J. H. Magdelijns, Larissa Schepers, Evelien Pijpers, Coen D. A. Stehouwer, Patricia M. Stassen

**Affiliations:** 1 Division of General Medicine, Department of Internal Medicine, Maastricht University Medical Centre, Maastricht University, PO Box 5800, 6202 AZ Maastricht, The Netherlands; 2 Section Clinical Geriatric Medicine, Division of General Medicine, Department of Internal Medicine, Maastricht University Medical Centre, Maastricht University, Maastricht, The Netherlands; 3 Division of General Medicine, Department of Internal Medicine, and Cardiovascular Research Institute Maastricht, Maastricht University Medical Centre, Maastricht University, Maastricht, The Netherlands; 4 Section of Acute Medicine, Division of General Medicine, Department of Internal Medicine, School of CAPHRI, Maastricht University Medical Centre, Maastricht University, Maastricht, The Netherlands

**Keywords:** Readmissions, Healthcare-related adverse events, Predictive factors

## Abstract

**Background:**

Readmissions are a burden for patients and increase healthcare costs. In Europe, factors associated with readmissions have not yet been extensively investigated. This study aimed to discover factors associated with readmissions in both young and older adult internal medicine patients. Furthermore, we explored the role of healthcare-related adverse events (AEs) in readmissions.

**Methods:**

All patients admitted through the emergency department to the internal medicine department in the last 2 weeks of each month (2011) were included. Information on index admissions and readmissions, defined as an unplanned admission within 30 days after discharge, was obtained from the electronic patient record system. Demographic, clinical, and organizational factors were evaluated for their association with readmissions.

**Results:**

Of all patients (*n* = 940), 17.3 % were readmitted; 16.9 % of the younger (<65 years, *n* = 485), and 17.8 % of the older patients (≥65 years, *n* = 455). Dependency in activities of daily living (ADL) was the only factor associated with readmissions in both all ages (OR 2.43) and in older patients (OR 3.19), while age was associated with readmissions in younger patients (OR 1.03 per year). AEs leading to 35.4 % of all index admissions were not associated with readmissions.

**Conclusions:**

Readmissions are common in medical patients, and, thus, remain a reason for concern in terms of patient safety and quality of care. AEs, responsible for to the index admission, were not associated with readmissions. ADL dependency was the only factor associated with readmission in patients of all ages and older patients, indicating that determining which patients are at risk for readmissions is not easy.

**Electronic supplementary material:**

The online version of this article (doi:10.1186/s40001-016-0230-0) contains supplementary material, which is available to authorized users.

## Background

Hospital readmissions (defined as an unplanned admission within 30 days after discharge) are not only highly prevalent (11.6–17.5 %) worldwide [1–5], but also a burden to patients, and they increase the already high healthcare costs. In addition, hospital readmissions are seen as a measure of quality of inpatient and post-discharge care [6, 7].

A better understanding of the factors related to readmissions is necessary to develop successful interventions. Some factors, such as race, the use of high-risk medications, comorbidities [Charlson comorbidity index (CCI)], and type of insurance, were found to be associated with readmissions [1, 3, 5, 8]. The studies that investigated these factors were mostly performed in the United States, with its specific healthcare structure and healthcare insurance, which make generalizability to settings in Europe difficult. One European study investigated the association between several factors and readmissions in a general medicine department and found that only the factor age (OR 1.01 per year) was independently associated with readmissions [9]. Therefore, in particular in Europe, information on factors related to readmissions is scarce.

Since the population is ageing, most hospitalized patients are, or soon will be, old. Older patients more often have comorbidities and disabilities, and they use more medications than younger patients. Multi-morbidity, polypharmacy, and the factor age per se [9] could make older patients more susceptible to hospital readmissions and AEs, which in their turn could lead to hospitalization [10–12]. However, the information on the factors that are associated with readmissions in older patients is as scarce as in patients of all ages and it is unknown whether or not these factors differ between younger and older patients, which we expect.

Readmissions are regarded as healthcare-related adverse events (AEs). It is known that other AEs than readmissions, e.g., medication-related AEs, are a common reason for hospitalization [12–16]. It may be possible that patients who have been hospitalized because of an AE are particularly vulnerable and thus more susceptible to readmissions. As far as we know, the role of AEs (that have led to hospitalization) in readmissions has not been studied before. In addition, it is unclear whether or not specific factors are associated with readmission in patients who were initially hospitalized because of an AE.

To address the aforementioned gaps in knowledge on factors related to readmissions, we aimed to identify factors, including not formerly investigated factors, such as AEs, which are associated with readmissions within 30 days after discharge in adult medical patients. Furthermore, we investigated which factors were associated with readmissions in younger (<65 years) and older patients (≥65 years). In addition, we analysed patients with a first (index) admission because of an AE to reveal factors that are associated with readmissions in this specific subgroup of patients.

## Methods

### Setting and study population

This study was conducted in secondary and tertiary university hospital (Maastricht University Medical Centre; MUMC+) in The Netherlands. Our hospital is a 700-bed teaching hospital, which is the only hospital of the city of Maastricht (≈120,000 inhabitants) and its surroundings, and which is the only university centre for the province of Limburg (≈1,119,000 inhabitants). The population of Maastricht is characterized by a high percentage of older patients: 18.1 % are 65 years or older. Annually, 22,000 patients visit our emergency department (ED), which is open 24 h, 7 days a week. In our hospital, all general internal medicine, endocrinology, oncology, haematology, nephrology, gastro-intestinal, and rheumatology patients presenting to the ED are assessed by internists specialized in acute care. General practitioners (GPs) refer the majority of patients (GP service is available 24 h/7 days). Some patients (notably high urgency patients) arrive by ambulance and a minority of patients are self-presenters. Almost all acutely patients admitted are presented through the ED (for more details on the organization of acute care in The Netherlands, see [17]). Every inhabitant of the Netherlands is obliged to have a health insurance, which ensures accessible healthcare for everyone.

All patients admitted through the ED to the department of internal medicine in the last 2 weeks of each month between January–December 2011 were included. Because of restricted availability of time, we had to limit our inclusion to half a year. By including the last 2 weeks per month of 1 year, we were able to study seasonal influences. Older patients were defined as patients aged 65 years or older, and younger adult patients as patients <65 years of age.

The first admission of a patient in our study period was considered the index admission. Exclusion criteria were: (1) (re-)admission to another department than internal medicine or transfer from another hospital, (2) elective or planned (re-)admissions, (3) already included in the study (every patient could only be included once), (4) death in-hospital during the index admission, and (5) death within 30 days after discharge unless the patient had already been readmitted prior to death.

### Data collection and definitions

Our hospital has an electronic patient record system, which gave us the opportunity to gather information from both the entire medical and nursing records. Admission charts and discharge letters were used to obtain patients’ age, sex, comorbidity, number of medications, living situation and functional status [cognitive function, performance in activities of daily living (ADL) and instrumental activities of daily living (IADL)], reason for admission, length of stay, and day/season of discharge. Comorbidity was calculated using the Charlson comorbidity index (CCI) [18, 19]. Living situation was categorized as community dwelling or living in a nursing facility. The performance in ADL and IADL was classified as independent or dependent based on information obtained by nurses during admission (Additional file 1: Table S1). Cognitive function was classified as normal or impaired. The following conditions were considered impaired cognition: dementia, mild cognitive impairment, intellectual disability, and/or delirium at time of admission. Reasons for the index admission were categorized as: gastro-intestinal, infection, malignancy, inflammatory, (auto-) intoxication, renal and/or electrolyte problems, allergy/anaphylaxis, syncope, cardiac, and other. Day of discharge was divided into weekday (Monday till Friday) or weekend day (Saturday and Sunday). Season of discharge was classified as: summer (June, July, August), autumn (September, October, November), winter (December, January, February), and spring (March, April, May). Information about readmissions was obtained by checking the electronic patient record system for admissions.

All index admissions were categorized as being caused by an AE or not. To define an AE, we used the following definition of the Dutch Internal Medicine Association: ‘Any event or state during or following treatment by a specialist or a general practitioner that influenced the health of the patient in such way that renewed treatment was necessary or that it led to damage’ [20]. Admissions because of problems we considered resulting from the progression of disease were not considered AEs. This definition is comparable to the definition used in other studies investigating AEs [21, 22]. AEs were divided into the following categories: medication-related, chemotherapy-related, diabetes mellitus-related, procedure-related, and other. To evaluate whether or not the admission was truly based on an AE, two independent researchers (FM and LS) evaluated the admission reason. In case of disagreement, a third independent researcher (EP or PS) [1, 23] decided on the issue. The four researchers followed an E-learning course on the identification of AEs. The group of patients with an index admission because of an AE will further be referred to as AE group.

A readmission was defined as an unplanned admission through the ED to the department of internal medicine within 30 days after discharge. The reasons for readmission were categorized in the same way as the reasons for index admission. The no readmission group consisted of the patients who were not readmitted within 30 days after discharge and who were alive.

### Statistical analysis

To investigate the factors that are associated with a readmission, we compared the readmission group with the no readmission group in the total study population (all ages), and in both younger and older patients. The same analyses were performed in the subgroup of patients with an index admission because of an AE. SPSS Statistics for Windows version 22.0 (SPSS Inc., Chicago, Illinois) was used to analyse all data. Data were shown as medians with ranges or numbers with percentages and odds ratios (ORs) with 95 % confidence intervals (95 % CI). Inter-group differences were compared using the Chi-square test or Fisher’s exact test for categorical data and the Mann–Whitney U test for continuous data. Cohen’s kappa was used to calculate the inter-rater agreement concerning AEs as reason for admission.

To evaluate which factors were related to readmission, we performed univariate and multivariate logistic regression analyses. Due to insufficient cases per admission reason category, this factor could not be included in the multivariate analyses. In addition, IADL was omitted out of the equation due to collinearity with ADL. In the multivariate analyses in the AE group of younger patients, cognition and living situation could not be included in the analyses due to insufficient cases. Age was included as a dichotomous variable (≤65 years (reference) vs. >65 years) in the analyses of the total study population. In the multivariate analyses in younger and older patients, age was included as a linear variable. CCI was included as a dichotomous variable: score of 0–2 (reference) vs. >2. The number of medications was categorized as follows: 0–5 (reference), 5–10, 10–15, and >15 medications. For season of discharge, we used summer as reference, for day of discharge, weekday was reference, and for sex, female was reference. *p* values below 0.05 were considered statistically significant.

The MUMC + Medical Ethics Committee approved this study.

## Results

### Total study population

**Fig. 1:**
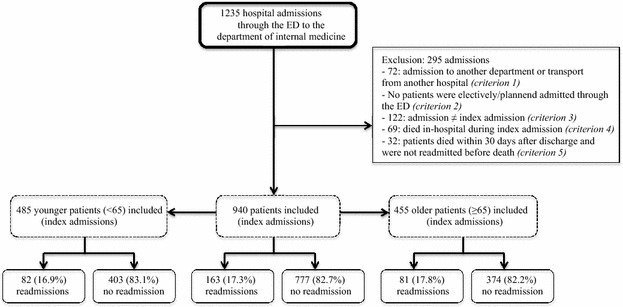
Flow chart. *ED* emergency department

In the study period, there were 940 index admissions (940 patients, Fig. 1). The readmission rate was 17.3 % and 72 patients (44.2 %) were readmitted for the same reason as their index admission. The median number of days until readmission was 10 (range 0–30, Table 1).

**Table 1:** Patient characteristics and inter-group differences

Study population	All patients				Younger patients (<65 years)			Older patients (≥65 years)		
*N* (%) or median (range)	Total study population	Readmission group	No readmission group	*p* value*	Readmission group	No readmission group	*p* value*	Readmission group	No readmission group	*p* value*
	*N* = 940	*N* = 163	*N* = 777		*N* = 82	*N* = 403		*N* = 81	*N* = 374	
Age, in years	64 (16–101)	65 (17–95)	64 (16–101)	0.39	*56 (17*–*65)*	*50 (16*–*65)*	*0.001*	76 (65–95)	77 (65–101)	0.21
Sex, female	493 (52.4)	77 (47.2)	416 (53.5)	0.14	39 (47.6)	214 (53.1)	0.36	38 (46.9)	202 (54.0)	0.25
CCI	*2 (0*–*11)*	*2 (0*–*9)*	*2 (0*–*11)*	*0.04*	*2 (0*–*8)*	*1 (0*–*11)*	*0.005*	2 (0–9)	2 (0–9)	0.96
Number of medications used^a^	5.0 (0–25)	5.0 (0–21)	5.0 (0–25)	0.70	4.0 (0–21)	3.0 (0–25)	0.11	*5.0 (0*–*16)*	*7.0 (0*–*20)*	*0.006*
ADL dependency, yes	44 (4.7)	12 (7.4)	32 (4.1)	0.08	2 (2.4)	9 (2.2)	1.0	10 (12.3)	23 (6.1)	0.05
IADL dependency, yes	55 (5.9)	11 (6.7)	44 (5.7)	0.59	2 (2.4)	11 (2.7)	1.0	9 (11.1)	33 (8.8)	0.52
Cognitive impairment^a^, yes	79 (8.4)	9 (5.5)	70 (9.0)	0.16	2 (2.4)	9 (2.2)	1.0	7 (8.6)	61 (16.3)	0.09
Living situation, nursing facility	65 (6.9)	9 (5.5)	56 (7.2)	0.50	1 (1.2)	7 (1.7)	1.0	8 (9.9)	49 (13.1)	0.58
Reason for index admission										
Gastro-intestinal	226 (24.0)	33 (20.2)	193 (24.8)	0.21	13 (15.9)	85 (21.1)	0.28	20 (24.7)	108 (28.9)	0.45
Infection	214 (22.8)	30 (18.4)	184 (23.7)	0.14	15 (18.3)	77 (19.4)	0.86	15 (18.5)	107 (28.6)	0.06
Malignancy	*167 (17.8)*	*54 (33.1)*	*113 (14.5)*	<*0.001*	*28 (34.1)*	*59 (14.6)*	<*0.001*	*26 (32.1)*	*54 (14.4)*	<*0.001*
Inflammatory	83 (8.8)	16 (9.8)	67 (8.6)	0.63	12 (14.6)	45 (11.2)	0.39	4 (4.9)	22 (5.9)	1.0
(Auto)-intoxication	*67 (7.1)*	*1 (0.6)*	*66 (8.5)*	<*0.001*	*1 (1.2)*	*64 (15.9)*	<*0.001*	0	2 (0.5)	1.0
Renal and/or electrolyte problems	49 (5.2)	9 (5.5)	40 (5.1)	0.85	5 (6.1)	16 (4.0)	0.38	4 (4.9)	24 (6.4)	0.80
Allergy/anaphylaxis	19 (2.0)	1 (0.6)	18 (2.3)	0.23	1 (1.2)	12 (3.0)	0.71	0	6 (1.6)	0.60
Syncope	8 (0.9)	0	8 (1.0)	0.36	0	4 (1.0)	1.0	0	4 (1.1)	1.0
Cardiac	8 (0.9)	1 (0.6)	7 (0.9)	1.0	0	2 (0.5)	1.0	1 (1.2)	5 (1.3)	1.0
Other	99 (10.5)	18 (11.0)	81 (10.4)	0.82	7 (8.5)	39 (9.7)	0.75	11 (13.6)	42 (11.2)	0.55
AE as reason for index admission	333 (35.4)	65 (39.9)	268 (34.5)	0.19	33 (40.2)	124 (30.8)	0.10	32 (39.5)	144 (38.5)	0.87
Length of stay, days	5 (1–131)	6 (1–66)	5 (1–131)	0.20	*6 (1*–*66)*	*4 (1*–*78)*	*0.01*	6 (1–36)	7 (1–131)	0.44
Day of discharge, day in weekend	182 (19.4)	33 (20.2)	149 (19.2)	0.75	20 (24.4)	84 (20.8)	0.48	13 (16.0)	65 (17.4)	0.77
Season of discharge										
Spring	251 (26.7)	42 (25.8)	209 (26.9)	0.77	17 (20.7)	107 (26.6)	0.27	25 (30.9)	102 (27.3)	0.51
Summer	243 (25.9)	47 (28.8)	196 (25.2)	0.34	24 (29.3)	109 (27.0)	0.68	23 (28.4)	87 (23.3)	0.33
Autumn	*234 (24.9)*	*29 (17.8)*	*205 (26.4)*	*0.02*	20 (24.4)	106 (26.3)	0.72	*9 (11.1)*	*99 (26.5)*	*0.002*
Winter	212 (22.6)	45 (27.6)	167 (21.5)	0.09	21 (25.6)	81 (20.1)	0.26	24 (29.6)	86 (23.0)	0.21

Numbers in italic show significant differences

*CCI* Charlson comorbidity index, *ADL* activity in daily living, *IADL* instrumental activity in daily living, *AE* adverse event

* *p* values are given for statistical tests between the readmission and the control groups

^a^Data of 2 patients were missing

In the readmission group, CCI was significantly higher (due to a skewed distribution, *p* = 0.04) and patients more often had an index admission because of a malignancy (33.1 vs. 14.5 %, *p* < 0.001) and were less often admitted because of an (auto-)intoxication (0.6 vs. 8.5 %, *p* < 0.001). Furthermore, patients discharged during autumn were significantly less often readmitted (*p* = 0.02) than when discharged during another season (Table 1). Index admissions because of an AE were equally prevalent in the readmission and the no readmission group.

Multivariate analyses in our total study population revealed that only ADL dependency was associated with readmissions (OR 2.43, *p* = 0.03) (Table 2).

**Table 2:** Factors associated with 30-day readmission in all, younger, and older patients

Possible factors (reference)	Multivariate analyses in all ages^b^				Multivariate analyses in younger patients (<65 years)^b^				Multivariate analyses in older patients (≥65 years)^b^			
	Total study population		AE group^c^		Total study population		AE group^c^		Total study population		AE group^c^	
	*N* = 940		*N* = 333		*N* = 485		*N* = 157		*N* = 455		*N* = 176	
	HR (95 % CI)	*p* value	HR (95 % CI)	*p* value	HR (95 % CI)	*p* value	HR (95 % CI)	*p* value	HR (95 % CI)	*p* value	HR (95 % CI)	*p* value
Age, in years^a^	1.12 (0.78–1.62)	0.53	0.97 (0.54–1.73)	0.91	*1.03 (1.01*–*1.05)*	*0.02*	1.01 (0.97–1.05)	0.61	0.98 (0.95–1.02)	0.37	1.02 (0.96–1.08)	0.51
Sex (female)	0.78 (0.55–1.11)	0.17	0.63 (0.35–1.11)	0.11	0.80 (0.49–1.31)	0.37	0.70 (0.31–1.58)	0.39	0.77 (0.46–1.27)	0.30	0.48 (0.20–1.15)	0.10
CCI (0–2)	1.45 (0.99–2.12)	0.06	1.31 (0.74–2.33)	0.35	1.59 (0.89–2.83)	0.12	1.84 (0.81–4.19)	0.15	1.16 (0.68–1.96)	0.59	0.90 (0.39–2.08)	0.81
Number of medications used (0–5)												
5–10	0.79 (0.53–1.19)	0.27	0.89 (0.47–1.68)	0.72	0.84 (0.45–1.56)	0.58	0.86 (0.35–2.14)	0.75	0.67 (0.39–1.18)	0.17	0.88 (0.35–2.20)	0.78
10–15	0.66 (0.34–1.25)	0.20	0.62 (0.25–1.54)	0.30	1.34 (0.50–3.62)	0.56	0.57 (0.13–2.47)	0.46	*0.40 (0.17*–*0.95)*	*0.04*	0.54 (0.16–1.83)	0.32
>15	0.63 (0.20–1.97)	0.42	0.87 (0.22–3.50)	0.85	1.35 (0.29–6.20)	0.70	1.28 (0.19–8.63)	0.80	0.22 (0.03–1.76)	0.15	0.48 (0.05–4.56)	0.52
ADL dependency	*2.43 (1.10*–*5.35)*	*0.03*	*7.05 (1.61*–*30.9)*	*0.01*	1.76 (0.25–12.4)	0.57	1.40 (0.12–16.9)	0.78	*3.19 (1.27*–*8.01)*	*0.01*	*10.0 (1.62*–*62.3)*	*0.01*
Cognitive impairment	0.54 (0.25–1.17)	0.12	0.26 (0.04–1.57)	0.14	0.74 (0.14–3.91)	0.72	–	–	0.45 (0.18–1.12)	0.09	0.24 (0.03–1.84)	0.17
Living situation (home)	0.67 (0.29–1.56)	0.36	0.24 (0.04–1.40)	0.11	0.41 (0.03–5.45)	0.50	–	–	0.91 (0.36–2.33)	0.85	0.25 (0.04–1.82)	0.17
AE as reason for the index admission	1.20 (0.83–1.75)	0.33	NA	NA	1.12 (0.65–1.95)	0.68	NA	NA	1.12 (0.66–1.91)	0.68	NA	NA
Length of stay (linear)	1.01 (0.99–1.02)	0.45	0.99 (0.96–1.03)	0.67	1.02 (0.99–1.04)	0.21	1.0 (0.94–1.06)	0.93	0.99 (0.96–1.02)	0.53	0.99 (0.95–1.03)	0.65
Season of discharge (summer)												
Spring	0.83 (0.52–1.33)	0.44	0.71 (0.32–1.55)	0.39	0.74 (0.37–1.48)	0.39	0.65 (0.21–2.03)	0.46	0.93 (0.48–1.81)	0.84	0.92 (0.29–2.91)	0.89
Autumn	0.60 (0.36–1.01)	0.05	0.50 (0.22–1.14)	0.10	1.03 (0.52–2.02)	0.94	0.62 (0.20–1.95)	0.41	*0.33 (0.14*–*0.77)*	*0.01*	0.42 (0.12–1.49)	0.18
Winter	1.10 (0.69–1.76)	0.69	0.84 (0.40–1.77)	0.66	1.12 (0.57–2.19)	0.75	0.86 (0.30–2.46)	0.78	1.01 (0.51–1.98)	0.98	0.82 (0.27–2.51)	0.83
Day of discharge (weekday)	1.10 (0.71–1.70)	0.66	1.46 (0.77–2.76)	0.24	1.27 (0.71–2.27)	0.42	1.26 (0.52–3.06)	0.61	0.93 (0.47–1.85)	0.85	1.59 (0.62–4.07)	0.33

Numbers in italic show significant differences

*HR* hazard ratio, *CI* confidence interval, *CCI* Charlson comorbidity index, *ADL* activity in daily living, *AE* adverse event, *NA* not applicable

^a^In the multivariate analyses of all ages, age was included as binominal covariate (≤65 and >65 years), in the multivariate analyses in younger and older patients, age was included as linear covariate

^b^IADL was omitted out of the equation due to collinearity with ADL. Because of insufficient cases, admission reasons could not be included in the analyses. In the AE group of younger patients, cognition and living situation could not be included in the analyses due to insufficient cases

^c^Logistic regression analysis performed in the subgroup of patients with an index admission because of an AE

### Younger and older patients

Of the younger patients, 16.9 % (*n* = 82) were readmitted after median 9 days. Age (56 vs. 50 years, *p* = 0.001) and CCI (2 vs. 1, *p* = 0.005) were significantly higher in the readmission group than in the no readmission group (Table 1). In the readmission group, patients more often had a malignancy (34.1 vs. 14.6 %, *p* < 0.001), but less often an (auto)-intoxication (1.2 vs. 15.9 %, *p* < 0.001) as reason for the index admission.

Multivariate analyses showed that age was associated with the early readmissions (OR 1.03 per year) (Table 2).

Of the older patients, 17.8 % (*n* = 81) were readmitted after median 11 days. In the readmission group, patients used fewer medications (median 5 vs. 7, *p* = 0.006), more often had a malignancy as reason for the index admission (32.1 vs. 14.4 %, *p* < 0.001), and were less often discharged during autumn (11.1 vs. 26.5 %, *p* = 0.002).

Multivariate analyses in older patients showed that ADL dependency was associated with readmissions (OR 3.19) (Table 2). Patients using 10–15 medications (OR 0.40), and being discharged during autumn (OR 0.33) were significantly less often readmitted.

### Patients with an index admission because of an AE

In the AE group (of all patients, 35.4 % of the study population), 65 patients (19.5 %) were readmitted compared with 98 patients (16.1 %, *p* = 0.19, data not shown) in the non-AE group. In the AE group, 29 patients (44.6 %) were readmitted because of the same reason as the index admission.

Patients in the readmission group less often had a medication-related AE, and more often a chemotherapy-related or a procedure-related AE than those in the no readmission group (Table 3). The inter-rater agreement for the judgment whether or not the admission was due to a healthcare-related AE was high (Cohen’s kappa: 0.82).

**Table 3:** Types of AE categories in readmission and no readmission group

Study population	All individuals				Younger individuals (<65 years)			Older individuals (≥65 years)		
*N* (%) or median (range)	Total study population	Readmission group	No readmission group	*p* value*	Readmission group	No readmission group	*p* value*	Readmission group	No readmission group	*p* value*
	*N* = 333	*N* = 65 (19.5)	*N* = 268 (80.5)		*N* = 33 (21.0)	*N* = 124 (79.0)		*N* = 32 (18.2)	*N* = 144 (81.8)	
Type of AE as reason for index admission										
Medication-related	*179 (53.8)*	*27 (41.5)*	*152 (56.7)*	*0.03*	*10 (30.3)*	*61 (49.2)*	*0.05*	17 (53.1)	91 (63.2)	0.29
Chemotherapy-related	*74 (22.2)*	*21 (32.3)*	*53 (19.8)*	*0.03*	*15 (45.5)*	*33 (26.6)*	*0.04*	6 (18.8)	20 (13.9)	0.58
Procedure-related	*50 (15.0)*	*15 (23.1)*	*34 (13.1)*	*0.04*	7 (21.2)	15 (12.1)	0.18	8 (25.0)	20 (13.9)	0.18
Diabetes mellitus-related	29 (8.7)	2 (3.1)	27 (10.1)	0.09	1 (3.0)	15 (12.1)	0.13	1 (3.1)	12 (8.3)	0.47
Other	1 (0.3)	0	1 (0.4)	1.0	0	0	NA	0	1 (0.7)	1.0

Numbers in italic show significant differences

*AE* adverse event

* *p* values are given for statistical test between the readmission and no readmission groups

Multivariate analyses of the AE group revealed that being ADL dependent was associated with readmissions (OR 7.05, Table 2).

Of the younger patients with an index admission because of an AE (*n* = 157), 33 (21.0 %) patients were readmitted. Of these, 17 (51.5 %) were readmitted for the same reason. Patients in the readmission group less often had a medication-related AE, and more often a chemotherapy-related AE than those in the no readmission group (Table 3).

In the multivariate analyses of the AE group of younger patients, no factors were found to be associated with readmissions (Table 2).

Of the older patients with an index admission because of an AE (*n* = 176), 32 patients (18.2 %) were readmitted, of whom 12 (37.5 %) were readmitted for the same reason. No statistically significant differences in the prevalence of the categories of AEs were found between the readmission and the no readmission group (Table 3).

Multivariate analyses in this AE group showed that ADL dependency was associated with readmissions (OR 10.0) (Table 2).

## Discussion

We found that 17.3 % (*n* = 163) of the medical patients admitted through the ED were readmitted within 30 days. ADL dependency was associated with readmissions in patients of all ages and in older patients (OR 2.43 and OR 3.19, respectively). Insightful inter-group differences were also found. In the readmission group of all ages and younger patients, CCI scores were higher than in the no readmission group. In both groups, more patients were readmitted when their index admission was due to a malignancy. Interestingly, being admitted because of an (auto-)intoxication was associated with fewer readmissions in all ages and in younger patients. No association was found between index admissions because of an AE and readmissions.

### Total study population

The 30-day readmission rate we found (17.3 %) is comparable with that of two other European studies [9, 24] and, despite of the differences in healthcare organization and insurance, to Asian [4, 25] and American studies [1, 3, 5, 8, 26]. This could indicate that the reasons for readmission lie in underlying diseases and/or patient characteristics rather than in the organization of the healthcare system. This hypothesis is supported by our and others’ findings that patients who were admitted because of a malignancy were more likely to be readmitted [4, 25].

ADL dependency was independently associated with readmissions, which was also shown in one other study [26], but not in another [3]. Furthermore, in the inter-group analysis, we, like others [3, 4], found CCI scores to be higher in the readmission than in the no readmission group. However, as CCI was not associated with readmissions in our multivariate analyses and as the association with ADL dependency was not a consistent finding in the literature, other factors could be associated with readmissions and should be investigated. Further, we could not confirm the finding in other studies that a longer length of stay [1, 3, 4, 8, 26] is associated with more readmissions. Furthermore, we like others [4, 5, 8] found no association between age and readmission, unlike others [1, 15, 26]. Finally, our hypothesis that patients with AEs are more vulnerable, and thus at higher risk of a hospital readmission could not be confirmed as admissions because of AEs were not associated with more readmissions.

### Younger and older patients

Readmissions occurred equally often in younger and older patients (16.9 and 17.8 %, respectively). As we expected, different factors were associated with readmissions in the two age groups. In younger patients, age (OR 1.03 per year) was associated with readmissions, but we did not find age to be important in older patients. We hypothesize that after reaching a certain age, age per se is less important. In older patients, ADL dependency was associated with readmissions (OR 3.19), which is in line with the findings of another study [2]. Two factors were found to be associated with fewer readmissions: using more medications (10–15 vs. 0–5, OR 0.40) and being discharged during autumn (OR 0.33). However, we only investigated 1 year and our finding could also be a ‘finding by chance’ and should thus be interpreted cautiously.

Until now, no factors are found to be consistently associated with readmissions, and therefore, it remains difficult to predict which patients are at risk for a readmission. However, our study suggests that interventions that aim to reduce readmissions should focus on older patients who are ADL dependent. In addition, as our study shows that readmissions occur just as often in younger, and in older patients, interventions should also focus on these younger patients, especially the ‘oldest’ younger patients.

### Subgroup of patients with an index admission because of an AE

In the AE group, patients were equally often readmitted as in the non-AE group. ADL dependency, again, was associated with readmissions (OR 7.05). Interestingly, patients less often had a medication-related AE in the readmission than in the no readmission group. An explanation could be that for medication-related AEs, a solution is available, for example, discontinuation of the drug or adjustment of dosage. This could make these patients less vulnerable for a readmission. On the other hand, patients more often had a chemotherapy-related AE in the readmission group than the no readmission group. Patients receiving chemotherapy often need prolonged and high-risk treatments, and are, therefore, more susceptible to readmissions. Finally, patients with procedure-related AEs leading to the index admission were more likely to be readmitted. We hypothesize that these patients are treated earlier, since specialists may be more cautious and readmit these patients more easily.

### Limitations

This study has some limitations. First, due to the retrospective nature of this study, data collection depended on documentation in the electronic record. However, this record includes not only medical, but nursing information as well, so little information is lost. Second, acute readmissions arranged through other ways than the ED were missed. However, in our hospital, almost all acute admissions take place through the ED. Furthermore, patients admitted to other hospitals are missed as well. However, the method used in this study is comparable with that of other studies [1, 4, 5], all patients are instructed to return to our hospital when (new) complaints evolve and, from our experience, only a few patients will go to another hospital. Furthermore, patients readmitted to other departments than internal medicine were not discussed. However, we only found 3 patients who were readmitted to other departments. Therefore, it is unlikely that our results were influenced by these readmissions. Third, our sample size is relatively small. Therefore, this study has less power to detect certain associations, and thus, associations could have been missed. Fourth, we focused on acute admissions through the ED to the department of internal medicine. Future research should also focus on readmissions after planned admissions and/or investigate factors associated with readmissions to other departments than internal medicine.

## Conclusions

Since readmissions are a serious burden to patients and considered an indicator of quality of care, it is important for hospitals to reduce readmissions. Moreover, as readmissions are common in medical patients, in both younger and older adult patients, they remain a great concern in terms of patient safety. We found that patients with a higher CCI or an index admission because of a malignancy, a chemotherapy-related AE or a procedure-related AE were more likely to be readmitted. However, index admissions because of AEs were not more frequently followed by readmissions than index admissions for other reasons. Furthermore, being ADL dependent was found to be a factor related to readmissions in both all ages and older patients. Thus, care (including post-discharge care) should be tailored to specific patient’s needs, with special attention to ADL dependency.

## Additional file

10.1186/s40001-016-0230-0 (Part of) Standard information obtained by nurses at admission.

## Abbreviations

ADL: activities of daily living
AE(s): adverse event(s)
CCI: Charlson comorbidity index
CI: confidence interval
ED: emergency department
GP: general practitioner
IADL: instrumental activities of daily living
MUMC+: Maastricht University Medical Centre
OR: odds ratio
