# Supplementary material for: Unplanned readmissions in younger and older adult patients: the role of healthcare-related adverse events
Source: Eur J Med Res. 2016 Sep 15;21:35. doi: 10.1186/s40001-016-0230-0 (PMC5025596; doi:10.1186/s40001-016-0230-0)
Supplement: Supplementary file 1 — 10.1186/s40001-016-0230-0 (Part of) Standard information obtained by nurses at admission. [file 40001_2016_230_MOESM1_ESM.docx]

| *Supplementary table S1. (Part of) Standard information obtained by nurses at admission* | |
| --- | --- |
| Do you need help with bathing/taking a shower? | yes/no |
| Do you need help with dressing? | yes/no |
| Do you need help with eating? | yes/no |
| Do you need help with using the bathroom? | yes/no |
| Do you need help getting out of bed or moving around? | yes/no |
| Do you have problems with urinating or defecation? | yes/no |
